# Gastric precancerous lesions:occurrence, development factors, and treatment

**DOI:** 10.3389/fonc.2023.1226652

**Published:** 2023-08-30

**Authors:** Yue Cao, Dongcai Wang, Guiyun Mo, Yinghui Peng, Zengzheng Li

**Affiliations:** ^1^ Emergency of Department, Yunnan Provincial Hospital of Traditional Chinese Medicine, The First Affiliated Hospital of Yunnan University of Traditional Chinese Medicine, Kunming, China; ^2^ Emergency Teaching and Research Department of the First Clinical School of Yunnan University of Traditional Chinese Medicine, Kunming, China; ^3^ Department of Hematology, The First People’s Hospital of Yunnan Province, Affiliated Hospital of Kunming University of Science and Technology, Kunming, China; ^4^ Yunnan Province Clinical Center for Hematologic Disease, The First People’s Hospital of Yunnan Province, Kunming, China; ^5^ Yunnan Blood Disease Hospital, The First People’s Hospital of Yunnan Province, Kunming, China; ^6^ National Key Clinical Specialty of Hematology, The First People’s Hospital of Yunnan Province, Kunming, China; ^7^ Yunnan Province Clinical Research Center for Hematologic Disease, The First People’s Hospital of Yunnan Province, Kunming, China

**Keywords:** gastric precancerous lesions, gastric cancer, traditional Chinese medicine, H.pylori, treatment

## Abstract

Patients with gastric precancerous lesions (GPL) have a higher risk of gastric cancer (GC). However, the transformation of GPL into GC is an ongoing process that takes several years. At present, several factors including H.Pylori (Hp), flora imbalance, inflammatory factors, genetic variations, Claudin-4, gastric stem cells, solute carrier family member 26 (SLC26A9), bile reflux, exosomes, and miR-30a plays a considerable role in the transformation of GPL into GC. Moreover, timely intervention in the event of GPL can reduce the risk of GC. In clinical practice, GPL is mainly treated with endoscopy, acid suppression therapy, Hp eradication, a cyclooxygenase-2 inhibitor, aspirin, and diet. Currently, the use of traditional Chinese medicine (TCM) or combination with western medication to remove Hp and the use of TCM to treat GPL are common in Asia, particularly China, and have also demonstrated excellent clinical efficacy. This review thoroughly discussed the combining of TCM and Western therapy for the treatment of precancerous lesions as conditions allow. Consequently, this review also focuses on the causes of the development and progression of GPL, as well as its current treatment. This may help us understand GPL and related treatment.

## Introduction

Gastric cancer is one of the most prevalent and lethal cancers in the world, particularly among older men. According to GLOBOCAN 2018 data, gastric cancer is the fifth most prevalent neoplasm and the third most lethal malignancy, accounting for an estimated 783,000 deaths in 2018 (1). The incidence and death of gastric cancer vary widely by geography and are greatly influenced by diet and Helicobacter pylori infection. Gastric atrophy, intestinal metaplasia, pseudopyloric gland metaplasia, and dysplasia during the pathological development of GC are called GPL (2). Multiple factors, including Hp, intestinal microbes, genetics (genetic susceptibility, epigenetics), and chronic inflammation, contribute to the development of GPL and GC (3, 4). Research on the causes of GLP and its progression to GC is at a lesser level than that on GC. However, dietary adjustment, smoking cessation, and exercise have the potential to prevent gastric cancer, whereas genetic testing enables earlier detection and, consequently, higher survival.

The eradication of H. pylori in the developing world remains a primary goal in the fight against gastric cancer due to its strong association with non-cardia gastric cancer. Conventional gastrectomy is associated with a high recurrence and death rate. However, improvements have been made in endoscopic screening and therapy, which have been confirmed in countries with a high incidence of GC, such as Japan and South Korea (5–7). Furthermore, TCM’s efficacy in reversing GPL has been confirmed by basic research, and it also shows excellent clinical efficacy. It is also used by many clinicians to eradicate Hp with or without western medicine. The biggest advantage of combining traditional Chinese medicine is to increase the clearance rate (including drug-resistant strains) and replace allergic or intolerant western medicine. For example, Licorice can be safely used in place of bismuth by patients (8).

Researchers are now investigating the mechanisms that lead to the emergence and continued development of GPL, and clinical studies have reported on the effectiveness, benefits, and drawbacks of several treatment regimens. Given these facts, we concentrated on the causes of GPL’s onset and growth as well as the advantages and disadvantages of existing treatment approaches.

## Occurrence and development factors

### Hp and imbalanced microbial flora

Chronic non-atrophic gastritis is mainly caused by Hp infection. Hp has been listed as a Class I carcinogen by the International Agency for Research on Cancer and the World Health Organization. Hp has been identified as the most common cause of sporadic GC and has the role of initiating the Correa cascade reaction. It is involved in the linear and progressive GPL process that is comprised of the final GC, gastric atrophy, gastrointestinal metaplasia, and chronic inflammation (9). The contribution of virulence factors is inseparable from Hp’s capacity to flourish in an acidic environment, cause persistent chronic inflammation of the stomach, and trigger the emergence of GPL and GC (10). These virulence factors mainly include urease, bacterial shape, flagellum number, and motility. These factors can aid HP in evading the host’s immune response, exposing it more directly to stomach cells, and inflicting a variety of host damage (10, 11). Macrophages generated from human monocytes downregulate the expression of miR-4270 during Hp infection, impair the expression and exposure of major histocompatibility complex class II (MHC-II) molecules on the plasma membrane, and diminish the antigen presentation ability (12, 13). In addition, Hp infection can activate NF-kappa B in the inflammation of gastric epithelial cells, stimulating the formation of chemokine IL-8 (14). Moreover, the development of the virulence factor CagA further interacts with MET receptors and promotes the proliferation of epithelial cells (15, 16). After infection, HP will recruit immune cells through cytokine production, leading to histological changes, and producing reactive free radicals that damage host DNA, thus inducing GC (17).

The GC tumor microenvironment (TME) is composed of tumor cells and matrix complexes that are constantly developing. The matrix is mainly composed of peripheral non-cancerous fibroblasts, epithelial cells, immune cells, blood cells, cytokines, growth factors, hormones, the extracellular matrix, and other extracellular components (14). Hp infection may damage the differentiation of M1 macrophages, induce M2 macrophages to differentiate or M1 to transdifferentiate into M2 macrophages and promote tumor progression and invasion by inducing angiogenesis in solid tumors and mediating immunosuppressive signals (15, 16). Moreover, HP can control macrophage function and affect TME by regulating specific microRNA (12). In addition, the production of arginase II (Arg2) in macrophages induced by Hp infection leads to cell apoptosis and inhibits the response of proinflammatory cytokines, thus promoting Hp immune escape (18, 19). Therefore, Hp can evade immune surveillance by impairing the antigen presentation of TAM or by disrupting the M1/M2 (or Mreg) balance to favor the M2 (or Mreg) phenotype (19, 20). The infiltration of immune cells in GC is significantly higher than that in GPL, especially the infiltration of macrophages. In GC, M2 phenotype Tumor-associated macrophage (TAM) can promote tumor proliferation and progression, which is related to the prognosis of the disease (21, 22).Single-cell sequencing also showed that macrophages play a key role in promoting gastric cancer (23). Furthermore, ErbB protein family receptors EGFR (ErbB1/HER1), ErbB2 (HER2/neu), ErbB3 (HER3), and ErbB4 (HER4) are activated after Hp infection. The next step is the activation of the phosphorylation cascade response in cells, which is an essential mechanism for cancer cell proliferation and survival (10, 24). The urease produced during the infection of patients contributes to the growth and metastasis of tumor cells by inducing angiogenesis (11). These findings suggest that by regulating gastric TME, HP can promote intestinal metaplasia and tumor transformation.

HP can control gastric acidity, which promotes bacterial growth and disrupts the flora in the stomach (25, 26). The increase in diversity and abundance of intestinal microbiota in patients with Hp infection has also been confirmed (27). Among them, the increase in the abundance of Propionibacterium acnes and lactic acid bacteria related to GC is unfavorable (28–30). Excessive propionibacterium acnes can promote the occurrence and development of GPL by producing proinflammatory cytokines (such as IL-15) (31). Lactic acid bacteria can promote the occurrence and development of GPL by producing N-nitroso compounds, and reactive oxygen species (32). These findings may indicate that HP is not a single pathogen that causes GPL and GC, and that HP is responsible for the ecological imbalance of the gut microbiota. However, studies on whether controlling gut flora (ecologically disturbed flora) can ameliorate or reverse GPL and GC are lacking. In addition to gastrointestinal flora, oral microbiota can promote the progress of GPL by inducing chronic inflammation, inhibiting the host immune system, anti-apoptotic activity, and producing carcinogens (volatile sulfur compounds, active nitrogen, and reactive oxygen species) (33). These results indicate that preventing Hp infection and maintaining the balance of microbial flora are clinically significant for the prevention and treatment of GPL and GC. In addition, when patients suffer from long-term or recurrent Hp infection, we should be vigilant that patients may develop GPL.

### Inflammatory factor

Chronic inflammation stimulates the infiltration of gastric epithelial cells by enhancing the expression of chemokines that promote the aberrant growth of DNA in diseased cells (34). During GPL, a large number of inflammatory cytokines are produced, which increases the chance of gene mutation and promotes the occurrence of GC. , IL-6, and tumor necrosis factor (TNF)- α are typically employed to assess the extent of gastric mucosal injury (35). IL-1β and IL-6 are common pro-inflammatory factors. A high concentration of IL-1β can cause an extensive inflammatory reaction, leading to tissue damage. IL-6 can induce tumor angiogenesis, regulate genes associated with the cell cycle, promote tumor proliferation, and regulate the local inflammatory environment to promote the tumor’s development. TNF-α maintains chronic inflammation, promotes the expression of other inflammatory cytokines, aggravates inflammation, and promotes tumor occurrence. Anti-inflammatory cytokines such as IL-4 and IL-10 are considered a double-edged sword in tumorigenesis (36). On the one hand, anti-inflammatory cytokines can reduce inflammation, yet inflammation and high expression of anti-inflammatory factors are major carcinogenesis risk factors. In contrast, anti-inflammatory cytokines can decrease the immunological response, thereby facilitating immune evasion (37, 38). From another perspective, inflammatory factors not only participate in the occurrence and development of GPL, but also help to indicate that patients have already developed GPL. Notably, the use of cytokines to diagnose diseases may necessitate the elimination of various interventions, including lung infections, sepsis, and other infections. Because the presence of exogenous microbes causes an aberrant rise in cytokines, particularly IL-6, IL-8, and IL-10 (39–41).

### Genetic changes

Chromatin remodeling-related pathways (centromeric chromatin remodeling and CENP-A containing nucleosome assembly) and their regulatory pathways (chromatin assembly or disassembly, nucleosome tissue, DNA packaging, and protein DNA complex assembly) are frequently altered in gastric cancer (GC) (42–44). However, there is an imbalance between low-grade intraepithelial neoplasia (LGIN) and high-grade intraepithelial neoplasia (HGIN) (45, 46). The pathway related to cell adhesion is the most affected in GC. HGIN gains the ability to invade and metastasize due to the disruption of the Rap1 signaling pathway and calcium-independent cells (47, 48). In the previously reported studies, the mutation of driving genes has been a source of concern for quite some time. Previous studies suggested that intestinal GC carcinogenesis did not follow any distinctive mutation pattern (49). In 2020, it was revealed that BCL2L11, RET, ALB, GRIN2D, and BRCA1 were the differentially expressed driving genes in GPL (LGIN+HGIN) and EGC. BCL2L11 is regarded as an apoptotic promoter, and its expression is markedly reduced in GPL. GRIN2D is regarded as an oncogene, and its expression is significantly elevated in GPL (50–52). These two genes may play a critical role in gastric tumorigenesis. This suggests that developing GRIN2D and BCL2L11 probes to diagnose GPL is a potential strategy. Furthermore, HP infection causes epigenetic changes in stomach mucosal abnormalities. According to multiple studies, CDKN2A, CDH1, and RUNX3 inhibit tumor suppressor genes in GPL patients infected with GC and Hp. Usually, aberrant DNA methylation causes the inactivation of the tumor suppressor genes, i.e., hMLH1 and CDH1 (53) (54),. Inactivation of the tumor suppressor gene is thus mostly induced by DNA methylation following Hp infection (55).

### Claudin-4

Claudin-4 has been reported to be highly expressed in GPL, GC, pancreatic cancer, esophageal cancer, ovarian cancer, endometrial cancer, bladder cancer, renal cancer, prostate cancer, and other solid tumors (56). In addition to its potential to cause disease directly, Hp is a high-risk factor for GPL because of how fast it can develop as a result of its chain reaction. In fused, non-transformed epithelial cells, Hp strain SS1 can reduce the expression of claudin-4, which raises cell bypass permeability and causes myosin light-chain (LC) phosphorylation in epithelial cells via activating myosin light-chain kinase (57). Hp lipopolysaccharide induces the initial activation of STAT3 and increases the expression of TLR2 in the cell membrane with the presence of Claudin-4 expression (58). Therefore, the expression of claudin-4, TLR2, and STAT3 is increased in GPL. Furthermore, aberrant upregulation of claudin-4 may produce epithelial-mesenchymal transition (EMT), intestinal metaplasia (IM), and GC, which is consistent with the high level of claudin-4 expression observed in PLGC and GC (59).

Claudin-4 overexpression is associated with promoter DNA hypomethylation during the development of GPL to GC. However, the decreased level of claudin-4 expression is related to the increase of DNA methylation during late GC (60). Thus, during the transition from GPL to GC, DNA methylation and Claudin-4 expression are inversely correlated. Claudin-4 knockdown also stimulates the PI3K/Akt pathway to accelerate GC cell motility, invasion, and proliferation (61). Additionally, similar to HP, the overexpression of claudin-4 can lead to the expression of IL-8, and the overexpression of IL-8 can promote the development of GPL to GC (62). Claudin-4 is a key factor in the progression of GPL to GC. These studies suggest that overexpression of claudin-4, or combined with high expression of IL-8, should be vigilant against the occurrence of GPL.

### Gastric stem cells

It might be the outcome of the multi-directional development of gastric stem cells from normal tissue to GPL and subsequently to GC (63, 64). Due to their natural capacity for self-renewal, stem cells have become the ideal candidate for transformation targets, which can contribute to the accumulation of genetic or epigenetic mutations essential for tumor development (65). Lgr5 gastric stem cells located at the bottom of the sinus can produce all types of epithelial cells. Therefore, gastric stem cells in this region may be the potential origin of GPL (66). The analysis of The Cancer Genome Atlas (TCGA) and the study of immunohistochemical staining showed that the intestinal adenocarcinoma at the junction of the gastric antrum and gastroesophageal was accompanied by the amplification of Lgr5. Furthermore, subsequent studies revealed that Lgr5 gastric stem cells progressed from microadenoma and gross adenoma to invasive intestinal GC, accompanied by the loss of Smad4 and Pten in the gastric antrum (67). In this view, the poor development of gastric stem cells could be one of the reasons for GPL. It is worth noting that detecting peripheral blood circulating tumor cells may be a useful tool for diagnosing GPL and GC (68).

### SLC26A9

Solute carrier family 26 members (SLC26A9) is a Cl− uniporter with very high expression levels in the gastric mucosa. SLC26A9 deletion resulted in the dysdifferentiation of stem and progenitor cells in an inflammatory milieu, leading to gastric cancer in mice. This may be related to the imbalance of multiple signaling pathways related to cell proliferation, apoptosis, and differentiation regulation as well as barrier integrity (69, 70). The absence of Slc26a9 expression leads to many molecular events, including β- Nuclear translocation of catenin and activation of the Wnt pathway leads to an imbalance of gastric epithelial cell proliferation and apoptosis, and tumorigenicity (71). Selective ablation of Slc26a9 in parietal cells also led to malignant transformation, although the pathological changes in the surface area occurred later than in intact Slc26a9 knockout mice. The study also found that the expression of Slc26a9 was gradually downregulated in human gastric diseases, from chronic gastritis to metaplasia, from GPL to early gastric cancer (72). Thus, the deletion of SLC26A9 shows significant tumorigenicity and hastens the progression of gastric cancer.

### Bile regurgitation

The concentration and duration of bile exposure are positively correlated with the incidence of GC and may lead to the recurrence of gastric stump cancer (73). A clinical study from China also showed that the independent risk factors of GC lesions included bile reflux grade, patient age, diet habits, and GC family history (73). Currently, the oncogenic mechanism of bile reflux is not fully understood, but it may be connected to the increased formation of reactive oxygen species (ROS) and reactive nitrogen species (RNS) induced by bile acid exposure that result in DNA damage and mutation of the oncogene p53 (74). Moreover, bile reflux can promote GC by activating the IL-6/JAK1/STAT3 pro-inflammatory signal pathway, and inhibiting STAT3 can reduce this carcinogenic effect (75). This provides a new approach to prevent bile reflux-associated GC.

### Exosomes

Exosomes can transport a variety of biological molecules, facilitating intercellular communication. Non-coding RNA (ncRNA) is a crucial regulator of complex communication between TME and GC cells and is one of the exosome’s most abundant contents. GC cell-derived ncRNA induces neutrophil autophagy and N2 polarization, while macrophages polarize into M2, promoting tumor development (76) and regulating GC cell drug resistance (76, 77). As a result, inhibiting the expression of GC-derived exosomes and ncRNA in exosomes may prevent the development of GC.

### microRNA

Spasmolytic polypeptide expression metaplasia (SPEM) is considered to be the precursor of intestinal metaplasia (IM) and GC. But little is known about the changes of miRNA during the development of IM and GC. MiR-30a is regarded as a negative tumor growth and metastasis regulator. Studies using mouse models and human samples revealed that miR-30a was downregulated in the epithelial cells of the SPEM, IM, and GC, indicating that this miRNA is essential for maintaining the homeostasis of the gastric gland (78, 79). In addition, miR-30a can inhibit the development of GPL and GC by targeting identified integrin α2 (ITGA2) in the stomach (80). As a result, miR-30a might be a useful therapeutic target to stop GPL and GC. In addition, miR-421 and miRNA-22-3p upregulated in the early stages of GPL and GC can be used as novel biomarkers for detecting GPL and early gastric cancer (81, 82). However, research is still needed to prove whether miR-421 and miRNA-22-3p are involved in the occurrence and development of GPL.

### Other

Lynch syndrome patients have a higher risk of GC than normal patients do, and MLH1 and MSH2(DNA mismatch repair genes) carriers also have a higher risk (83) (84). A recent prospective study demonstrated that 11 essential lipids including eight phospholipids and 3 free fatty acids (FFAs) are negatively related to the risk of GPL progression and that the characteristics of lipomics may be associated with the risk of gastric disease progression and GC. Earlier studies have also demonstrated the link between lipids and GC. The occurrence and development of GC tumors will be impacted by the phospholipid content of membrane fluidity and signal transduction (85–87). APOA1BP, PGC, HPX, and DDT were discovered to be connected to the risk of gastric disease progression and to be able to forecast the progression of gastric disease in a prospective study on proteomic analysis, GPL, and early gastric cancer (88). Homocysteine (Hcy), vascular endothelial growth factor (VEGF) and serum gastrin 17 (G17) can exhibit different levels of expressions in precancerous lesions. They are also highly expressed in gastric cancer. Besides, they are involved in the occurrence and development of gastric cancer and can be regarded as crucial indexes with clinical significance for the differential diagnosis of gastric cancer and precancerous lesions in the early stage (89). In addition, environmental factors living habits, immunity, heredity, age, and high salt and low vitamin diet are also related to the occurrence and development of GPL.

### Diagnostic recommendations

Most GPL patients have no obvious symptoms, often leading to delays in diagnosis and treatment. The above factors miRNA-30a/421/22-3p, Hcy, VEGF, G17, anti-Hp IgG have been proved to be Biomarker of GPL, and may be helpful to the discovery of GPL. In addition, scholars have also reported other indicators that can help diagnose GPL. The increase in anti-Hp IgG levels may help evaluate and manage gastric precancerous lesions (90).The microsatellite facility (MSI) gradually increases from GPL to GC. The early detection of MSI may be a warning indicator for GPL (91). Serum pepsinogens, serum gastrin and Hp Cag A status are important tests in detecting GPL (92). The joint detection of Monoclonal gastric cancer 7 antigen (MG7-Ag) and cyclo-oxygenase 2(COX-2) can predict the occurrence of GPL (93). However, some indicators may only provide clues and cannot directly serve as a diagnostic basis. Although Hp infection, bile reflux, and abnormal expression of inflammatory factors are factors that affect the occurrence and development of GPL, poor specificity and sensitivity may only have a warning effect. Genetic changes, exosomes, microRNA, and tumor cell monitoring are usually not routine testing methods for GPL. We suggest that examination of these markers be conducted under conditions, and further endoscopic diagnosis should be used when patients are found to have long-term or recurrent Hp infection, bile reflux, and abnormalities in the aforementioned markers. In the endoscopic diagnosis, antifoam and mucolytic agents should be used to improve the visibility of Gastric mucosa. Appropriate spasmolysis treatment in patients with severe gastric motility can improve the observation field of vision (94). At the same time, Application of magnifying narrow band imaging endoscopy can be used to detect early gastric cancer and GPL, which is superior to conventional endoscopic examination in the diagnosis of early gastric cancer and GPL (95). In addition, as a promising new screening method, Magnetic controlled capsule endoscopy. It is used to diagnose gastrointestinal diseases in Asymptomatic individuals, especially GC and GPL, with the advantages of safety, non-invasive, high efficiency and cost-effectiveness (96). Although endoscopic diagnostic techniques have been improved, biopsy and pathological diagnosis should also be performed for suspicious and visible lesions. In addition, we believe that the development of relevant target probes should also be developed. As mentioned earlier, BCL2L11 is significantly reduced in GPL, while GRIN2D, Claudin-4, APOA1BP, PGC, HPX, and DDT are significantly increased in GPL. Therefore, developing relevant probes for detecting biopsy tissue may greatly improve the accuracy of GPL diagnosis.

## Treatment

### Eradicate Hp

Eradicating Hp can prevent peptic ulcers, and GPL and GC are well-known. Currently, tetracycline, bismuth agent, metronidazole, and quadruple PPI are frequently used in clinical settings. But in recent years, HP has manifested a high infection rate, a high pathogenicity, a high level of antibiotic resistance, and a low eradication rate in many areas. In many nations and regions, large-scale and ineffective eradication programs are being used. Still, Hp resistance (antibiotic resistance mutation) continues to rise, metronidazole resistance is about 80%, and in some areas, levofloxacin and clarithromycin resistance is over 60% (97). Due to the influence of Hp globular transformation and host CYP2C19 gene polymorphism, the eradication rate of Hp decreased from 90% to 70% (98, 99). In other words, the biggest issue with treating HP infection is antibiotic resistance. Utilizing proton pump inhibitors to reduce gastric acid secretion, raise gastric pH, and thereby increase the effectiveness of antibiotics is a crucial method in addition to using the proper treatment plan for each treatment.

Additionally, choosing the right PPI based on a patient’s CYP2C19 genotype can raise the eradication rate (98). However, long-term acid suppression therapy can lead to three side effects in Hp-positive patients, including the development of other microorganisms, an increase in cytokine levels, and an increased risk of atrophic gastritis (100). The use of proton pump inhibitors (PPI) before diagnosing autoimmune atrophic gastritis can significantly increase the risk of GPL development (101). Therefore, regulating the use of acid suppressants will be more conducive to preventing and treating GPL.

Recently, the newly synthesized silver super nanoclusters have been used alone and in combination with metronidazole to treat Hp infection and achieved good results (10). In patients with clarithromycin-resistant strains of Hp, a prospective study demonstrated that adding bismuth to the conventional triple therapy can be used as the first-line treatment strategy. Bismuth is suggested to be used as a first-line treatment, particularly in regions where clarithromycin tolerance is high). It should be noted that smoking will increase the failure rate of Hp eradication treatment (except for Vonoprazan treatment) (102). Elevated vitamin D levels may help eradicate Hp (103). Furthermore, quintessence, a Chinese medication has a unique advantage in eliminating Hp.

The eradication of Hp after early cancer resection can prevent GC. However, eradication cannot prevent the development of new cancers but can only prevent cancer in patients without GPL (atrophy, IM, and dysplasia) (104). In addition, recent studies have shown that eradicating Hp may increase the risk of other diseases and increase antibiotic resistance (10). The risk of a series of chain reactions following a Hp infection is much greater than the risk of eradication, even though other risks are associated with eliminating Hp, as was already mentioned. Consequently, the eradication of HP should continue to be the primary consideration.

### Endoscopic resection

Early gastrointestinal tumors are now treated with endoscopic organ preservation instead of surgery. Endoscopic resection (ER) includes polypectomy, endoscopic mucosal resection, and endoscopic submucosal dissection. Several societies have recommended the standard indications for endoscopic resection of GPL and intestinal GC. Endoscopic evaluation and monitoring should be carried out for patients with genetic susceptibility to GC (105). Low-grade dysplastic gastric mucosal lesions with a diameter of less than 1 cm can be treated with either Endoscopic mucosal resection (EMR) or endoscopic submucosal dissection (ESD), but ESD is better for patients with large lesions and those who have high-grade dysplasia (HGD) or early gastric cancer (EGC) (106). Countries with a high incidence of GC have implemented systematic screening programs and demonstrated the benefits of early detection and endoscopic resection of GPL and EGC, which can provide curative treatment and significantly reduce the recurrence rate and incidence (6, 7). In Japan, ER has become the treatment standard for EGC and GPL (107). However, there are still some problems with ER technology. For instance, it is only used for tumors with a very low risk of lymph node metastasis that is suitable for total resection. Additionally, data collection in regions with a low incidence rate is lacking. This also provides unlimited opportunities and challenges for the future promotion of ER technology.

### COX-2 inhibitors

Based on the relationship between chronic inflammation and cancer, non-steroidal anti-inflammatory drugs (NSAIDs) are well-known cyclooxygenase (COX) inhibitors for the treatment and prevention of cancer. COX is divided into two different subtypes, i.e., COX-1 and COX-2 (108). Prostaglandins produced by COX-1 play a role in platelet function and gastrointestinal cell protection, while prostaglandins produced by COX-2 are involved in pain and inflammation (109). It is believed that NSAIDs’ therapeutic impact is due to their inhibition of COX-2; however, this action is not specific to COX1/2. As a result, inhibiting COX-1 is a common side effect of NSAID therapy, especially in the gastrointestinal tract (110) (39). Therefore, when using NSAIDs in clinical treatment, care should be taken to avoid the side effects caused by COX-1 inhibition.

### TCM treatment

Chinese medicine is used extensively to prevent and treat GPL in Asia, especially in China, due to the rising drug resistance, adverse side effects of antibiotic therapy, and high rate of recurrence of endoscopic treatment (111, 112). Since chronic GPL inflammation permeates the entire process, anti-inflammatory medicine is crucial for treating GPL and GC. In rats with chronic atrophic gastritis and precancerous lesions, weiqi decoction can reduce COX-2 and increase PGE2, inhibiting gastric inflammation (113). Manpixiao Decoction can prevent the progress of GPL by improving systemic inflammation in local gastric mucosa and inhibiting EGFR-PI3K-AKT-related EMT pathways (114). *Banxia Xiexin* decoction can hinder the signaling path of the toll-like receptors (TLRs)/nuclear factors- κ B(NF- κ B). *Huangqi Jianzhong* Decoction combined with acupuncture can reduce high-sensitivity C-reactive protein, IL-6, TNF- α, and Pepsinogen (PG) II level (8). This suggests that Manpixiao decoction, *Banxia Xiexin* decoction, and Huangqi Jianzhong Decoction may have a specific therapeutic effect on inflammation in GPL.

Berberine (BBR) is a quaternary ammonium alkaloid extracted from Coptis Chinensis, which has anti-inflammatory, anti-cancer, anti-ulcer, antibacterial, and immune-enhancing activities. The study found that BBR has multiple effects on the cascade reaction of Correa, which can prevent and treat GPL and GC and even reverse the development process from GPL to GC in some cases (115). By controlling inflammatory cytokines, promoting apoptosis, controlling macrophage polarization, and controlling autophagy during GPL, BBR can reverse mucosal atrophy and avoid intestinal metaplasia and hyperplastic lesions. Additionally, BBR has a therapeutic effect on GC by primarily preventing cell migration, proliferation, and angiogenesis, and it can make chemotherapy drugs more sensitive (115). Additionally, BBR combined with conventional triple therapy can increase the effectiveness of treating clinical symptoms, eradicating HP, and minimizing adverse side effects (116). These data demonstrate that BBR in Coptis Chinensis has a therapeutic impact on pathological tissue changes, immune disorders, cell proliferation, and migration in GPL.

Additionally, it can aid in the eradication of HP and improve the sensitivity of chemotherapy medications. Baicalin and breviscapine also inhibited the sulfhydryl group surrounding the Hp urease active site, particularly Cys321, in a non-competitive manner (117). These two active ingredients of Chinese herbal medicine may be candidates for urease inhibitors to treat Hp infection. Licorice is commonly used as a restorative material. Glycyrrhizic acid, its main component, has a positive correlation effect on preventing HP growth (118). The clinical trial discovered that adding licorice to the triple therapy based on clarithromycin could boost the eradication rate of Hp (8, 119). Licorice has also increased the expression and secretion of HP-related vascular endothelial growth factor (VEGF), as well as cyclooxygenase 2 (COX-2), TNF- α, and inducible nitric oxide synthase (iNOS) (120).

Aerobic glycolysis provides energy for tumor cell growth. New research shows that aerobic glycolysis is already very active in the GPL stage (121–123). This suggests that intervention in glycolysis at this stage may delay or halt GPL development. A reported study on the animal model suggested that astragaloside IV could reverse the N-methyl-N-nitro-N-nitroso-induced GPL in rats because astragaloside IV could inhibit glycolysis through the dual regulation of p53/miRNA-34a/LDHA and p53/TIGAR signal pathways (124, 125). Dendrobium officinale polysaccharide (DOP) may be a potential candidate drug for GPL. Studies have found that DOP can inhibit the development of GPL by up-regulating the expression of PER3 and AQP4 genes and proteins (126).

ROS produced by gastric immune cells and epithelial cells will damage gastric mucosa, causing irreversible damage to protein, cell lipid, and DNA, leading to GPL and eventually GC (127, 128). Ginkgo biloba extract has been shown in prior studies to increase superoxide dismutase activity and decrease malondialdehyde concentration in GPL rats, indicating that it has protective effects against oxidative stress (129). In addition, this study also confirmed that *Ginkgo biloba* could block the progress of GPL by regulating cell proliferation and apoptosis. Recently, it has been used to treat GPL and GC by inducing apoptosis and inhibiting cell proliferation (121, 130, 131).

When treating Hp-positive gastritis, Liujunzi decoction and quadruple therapy were more effective than quadruple therapy alone. Compared to the triple therapy alone, the combination of Yiwei decoction and the treatment can help HPAG patients to lower their serum inflammatory factor levels. Fuzheng Qingre Qingtang combined with triple therapy is more effective than triple therapy alone in treating chronic superficial gastritis brought on by Hp infection (8).

### Other treatments

A recent study found that daily dosages of 1.5 mg, 100 mg, and 10 mg of vitamins A, C, and E, respectively, could effectively lower the incidence of GPL by about one-third (132). However, high doses of vitamin A and vitamin E supplements were associated with increased GC mortality. In contrast, the risk of death from GC was found to be lowered in a trial including the use of low-dose dietary vitamins (physiological rather than pharmaceutical dosages) (133). A recent study on the reversal of GPL with folic acid supplementation found that folic acid can help heal atrophic gastric mucosal lesions and reverse intestinal metaplasia in GPL patients (134).

### Viewpoint

**Figure 1:**
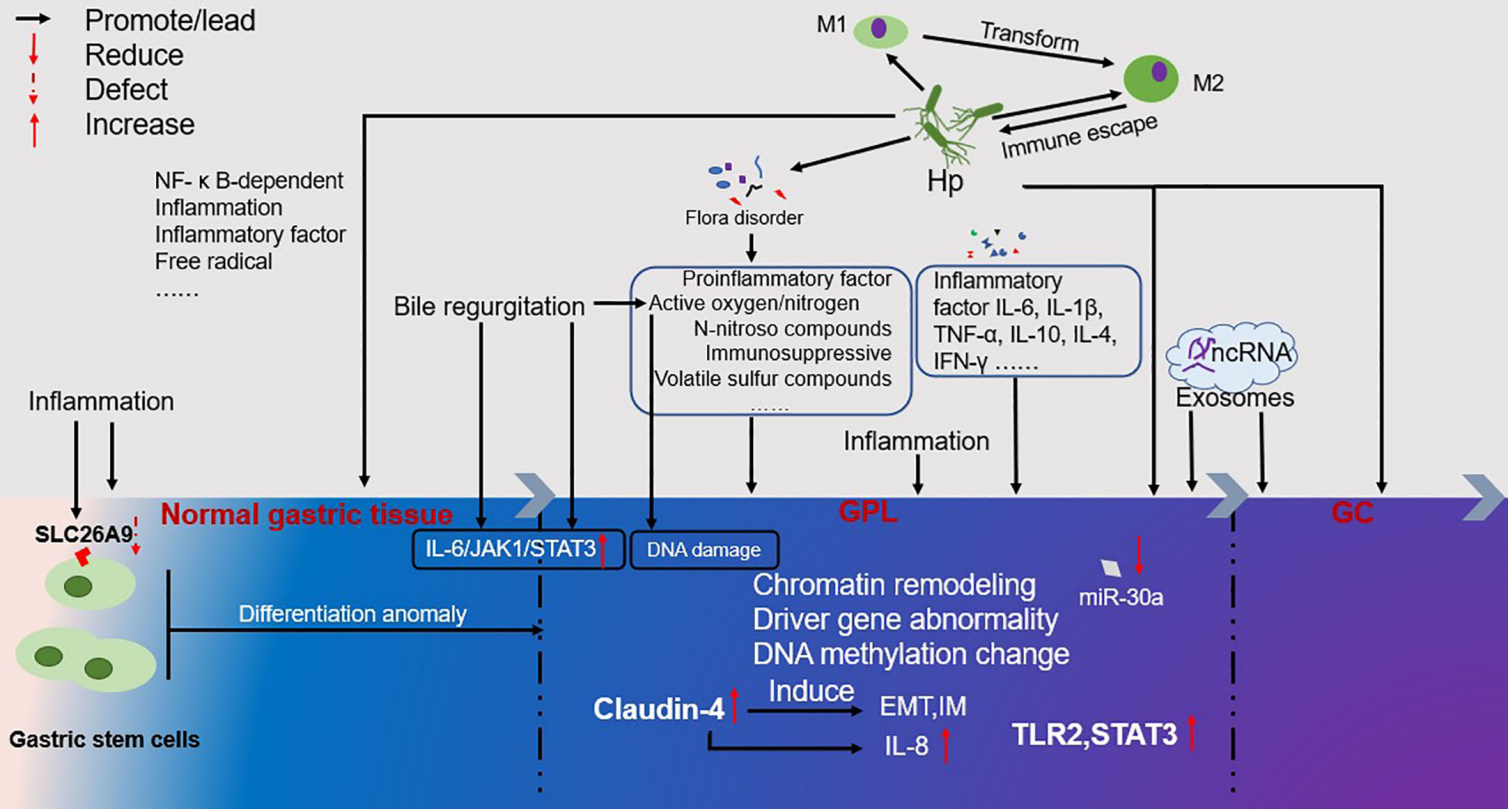
Overview of the occurrence and development of GPL. The color change from left to right represents the process of GPL development. Inflammation runs through the occurrence and development of GPL. At the same time, it will lead to the loss of gastric stem cells SLC26A9, which will lead to the occurrence of GPL and GC. The multi-directional differentiation of gastric stem cells may also be the source of GPL and GC. Inflammatory factor IL-6, IL-1 in patients with GPL β, TNF- α, IL-10, IL-4, IFN- γ The formation of GC will be accelerated. Bile regeneration will cause the occurrence and development of GPL through the IL-6/JAK1/STAT3 signal pathway, and the active oxygen/nitrogen generated will cause DNA damage. The high expression of Claudin-4 will induce the formation of EMT and IM, and the induced IL-8 will accelerate the formation of GC. The ncRNA in Exosomes can also promote the development of GPL and the formation of GC. From the occurrence of GPL to the formation of GC, under the influence of various factors, chromatin modeling, driver gene abnormality, DNA metabolism change, etc. are phenomena that occur in this process, but they are also the driving force for GPL to develop towards GC.

Complex factors contribute to the occurrence and development of GPL as GC. Herein, the current study summarizes the findings of various reported studies. Furthermore, this study summarizes the factors associated with GPC occurrence and development as follows: 1. Infection with *H. pylori* initiates the Correa cascade and induces chronic inflammation while simultaneously affecting the balance of M1/M2 in the local stomach, resulting in immune dysfunction. In the process of infection, it evades the host immune response and destroys cell DNA through virulence factors, regulates the microenvironment, and promotes the occurrence and transformation of GPL into tumors. Moreover, it causes the imbalance of microbial flora and the production of other harmful carcinogens, such as N-nitroso compounds, active oxygen, and volatile sulfur compounds, thus causing the occurrence and development of GPL. 2. Multidirectional differentiation of gastric stem cells could be one cause of gastric disorders. 3. Long-term exposure to highly reactive oxygen species (including bile reflux, microbial virulence factors, related gene changes, chronic inflammation, etc.) promoted the occurrence of GPL and accelerated the formation of GC. 4. Chromatin remodeling, abnormal DNA methylation, and aberrant expression of driving genes are not only the manifestations of GPL but also its driving factors. Perhaps the continuous process from normal tissue to GPL to GC is a chain reaction caused by the destruction of the normal microenvironment. Characteristics of the microenvironment of GPL include chromatin remodeling, abnormal DNA methylation, abnormal expression of driving genes, oxidative stress, imbalance of flora, persistent inflammation, production of harmful substances, immune disorders, changes in cell function, hypermetabolism, and the generation and proliferation of diseased cells and tissues. In such an unfavorable microenvironment, GPL develops into GC (**Figure 1**).

**Figure 2:**
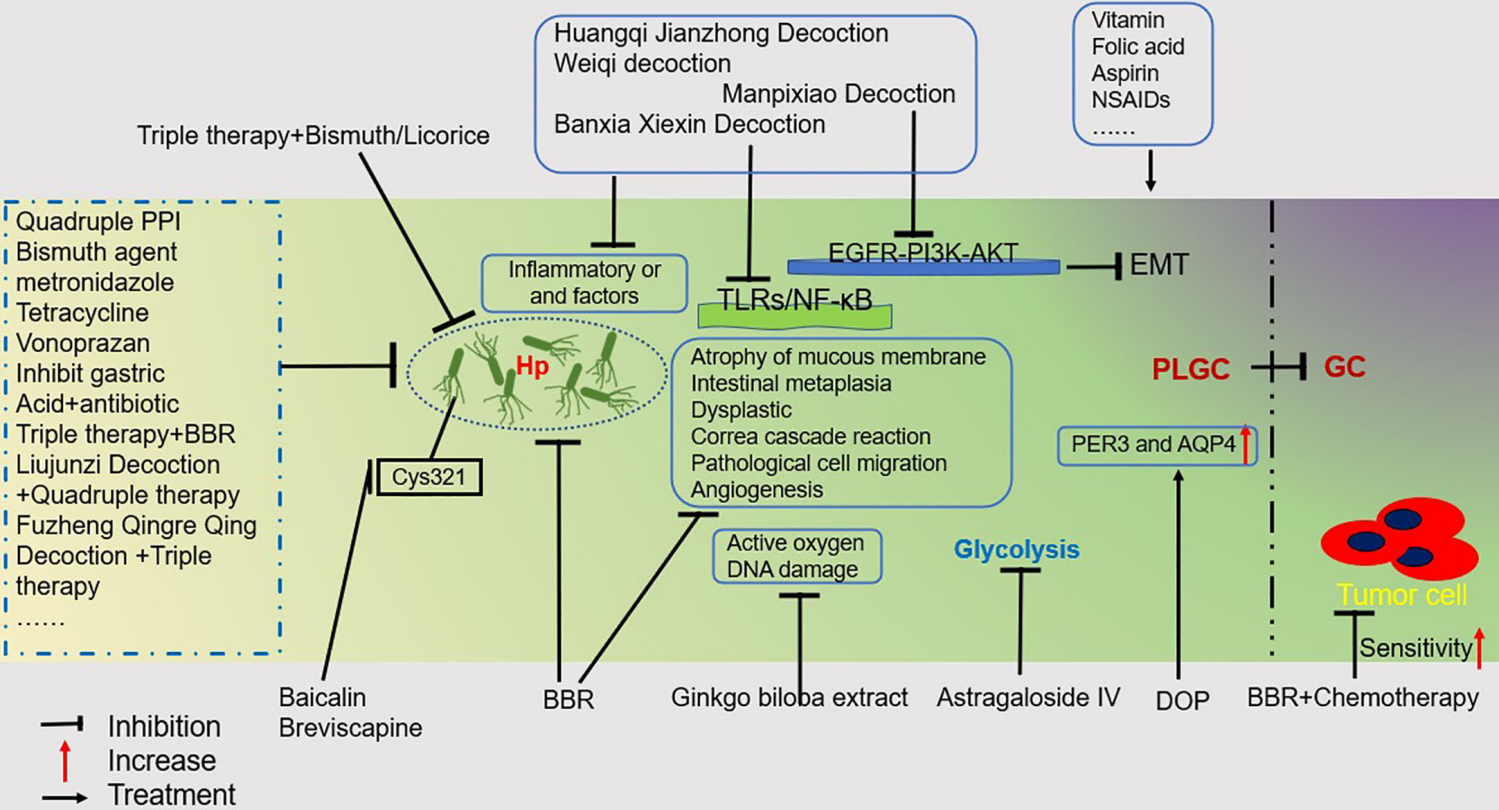
GPL Treatment Overview. This figure illustrates some drugs or regimens that can help treat PLGC. Mainly including traditional Chinese medicine, Western medicine, and a combination of traditional Chinese and Western medicine treatment plan for inhibiting Hp;Therapeutic drugs that inhibit Inflammatory or and factors; Drugs that inhibit DNA damage caused by reactive oxygen species; Drugs that inhibit glycolysis in PLGC; Drugs that treat PGLC by upregulating PER3 and AQP4; By inhibiting TLRs/NF- κ B pathway and EGFR-PI3K-AKT pathway to treat PGLC drugs; A regimen that helps increase the sensitivity of tumor cells to chemotherapy.

The intervention of GPL has important clinical significance in blocking the development of GC. TCM is a profound and ancient healing method that dates back more than 3,000 years. The discipline of TCM combines profound philosophical wisdom with thousands of years of Chinese medical practice and understanding. Here we only list some reported Chinese herbal formulations and active ingredients of Chinese herbal medicines. To prevent and alleviate GPL, TCM focuses on eliminating Hp, reducing inflammation, regulating the proliferation and apoptosis of gastric mucosal epithelial cells, inhibiting oxidative stress, decreasing glycolysis, inhibiting angiogenesis, and regulating immunity (**Figure 2**). However, this does not fully reflect the therapeutic principles of TCM because it needs to carry out “Bianzheng” treatment for patients. Additionally, the eradication of Hp is the first method to be considered. As drug resistance among Hp patients rises, treatments that combine TCM and western medicine are proving more successful. On the one hand, it can increase the clearance rate, and on the other, it can replace ineffective drugs and lessen side effects. We strongly suggest that TCM be used along with western medicine to treat GPL and GC. Taken together, the incidence of GPL and the development of GC have both been the subject of extensive scientific study, but there is still much remaining elusive to understand about both phenomena. Although Western medicine, alone or in combination, can provide significant results, more research is needed to explain specific therapeutic mechanisms. Research on TCM toxicity concerns should be prioritized, and new analytical techniques and procedures should be employed to examine the toxicity aspects of TCM. TCM often contains multiple ingredients, with the dosage constantly adjusted according to the patient. Furthermore, many studies are only published in their respective regions. Chinese scholars need to figure out how to take a successful “China plan” to a global scale. Well-designed clinical trials and experimental studies should be conducted to aid in a better understanding of TCM’s mechanism and to promote its modernization in the treatment of GPL.

## Author contributions

YC wrote manuscripts and drawings. DW and GM collected documents and revised language logic. YP corrected manuscripts and drawings and participated in the design of manuscripts. ZL designed the overall content of the article, provided funding support and summarized the article. All authors contributed to the article and approved the submitted version.
